# Species-Specific Unbound Fraction Differences in Highly Bound PFAS: A Comparative Study across Human, Rat, and Mouse Plasma and Albumin

**DOI:** 10.3390/toxics12040253

**Published:** 2024-03-29

**Authors:** Sangwoo Ryu, Woodrow Burchett, Sam Zhang, Seyed Mohamad Sadegh Modaresi, Juliana Agudelo Areiza, Emily Kaye, Fabian Christoph Fischer, Angela L. Slitt

**Affiliations:** 1 Department of Biomedical and Pharmaceutical Sciences, University of Rhode Island, Kingston, RI 02881, USA; ryus05@uri.edu (S.R.); smodaresi@uri.edu (S.M.S.M.); juliana_agudelo@uri.edu (J.A.A.); emilykaye@uri.edu (E.K.); 2 Pharmacokinetics, Dynamics and Metabolism, Pfizer Worldwide Research & Development, Pfizer Inc., Groton, CT 06340, USA; woodrow.burchett@pfizer.com (W.B.); sam.zhang@pfizer.com (S.Z.); 3 Harvard John A. Paulson School of Engineering and Applied Sciences, Harvard University, Cambridge, MA 02138, USA

**Keywords:** PFAS, equilibrium dialysis, protein binding, toxicology

## Abstract

Per- and polyfluoroalkyl substances (PFAS) are a diverse group of fluorinated compounds which have yet to undergo comprehensive investigation regarding potential adverse health effects and bioaccumulative properties. With long half-lives and accumulative properties, PFAS have been linked to several toxic effects in both non-clinical species such as rat and mouse as well as human. Although biological impacts and specific protein binding of PFAS have been examined, there is no study focusing on the species-specific fraction unbound (f_u_) in plasma and related toxicokinetics. Herein, a presaturation equilibrium dialysis method was used to measure and validate the binding of 14 individual PFAS with carbon chains containing 4 to 12 perfluorinated carbon atoms and several functional head-groups to albumin and plasma of mouse (C57BL/6 and CD-1), rat, and human. Equivalence testing between each species-matrix combination showed positive correlation between rat and human when comparing f_u_ in plasma and binding to albumin. Similar trends in binding were also observed for mouse plasma and albumin. Relatively high Spearman correlations for all combinations indicate high concordance of PFAS binding regardless of matrix. Physiochemical properties of PFAS such as molecular weight, chain length, and lipophilicity were found to have important roles in plasma protein binding of PFAS.

## 1. Introduction

Per- and polyfluoroalkyl substances (PFAS) constitute a large group of manufactured chemicals that possess unique physical and chemical properties which make them desirable for commercial and residential uses, such as resistance to oil, water, heat, and biodegradation [1,2]. These characteristics have made PFAS broadly used for numerous applications including firefighting foams, textiles, nonstick cookware, fast food packaging, and cosmetics. PFAS use has expanded since their introduction in the 1940s, and it is estimated that there are 10,000+ PFAS present in the environment. Because most PFAS are highly persistent, production and use have led to extensive environmental contamination [2,3,4,5,6,7]. Exposure to certain PFAS (e.g., perfluorooctanoic acid [PFOA] and perfluorooctanesulfonic acid [PFOS]) has been linked to various health issues, including kidney and testicular cancer [8], decreased response to vaccination [9,10], reduced birth weight [11,12,13], elevated serum cholesterol levels, and hypothyroidism [14]

PFOS and PFOA are long carbon-chain PFAS, which are considered to be “legacy PFAS”. They are of particular toxicological concern because they are highly persistent in the environment and bioaccumulate in living organisms despite having been phased out of production [15,16,17]. PFOS and PFOA are thought to be bioaccumulative, in part, because they do not undergo extensive metabolism and breakdown by living systems. For example, NHANES data from 2017–2018 revealed that PFOA and PFOS are detected in over 99% of the general population sampled, indicating their widespread presence at measurable levels among the US population [18,19]. In humans, the bioaccumulation of certain PFAS is extensive—with elimination half-lives ranging from weeks to years [20,21]—and is generally dependent on carbon chain length. Data shows that PFAS with long perfluorinated carbon chains such as PFHxS (*η*_pfc_ = 6), PFOA (*η*_pfc_ = 7), PFOS (*η*_pfc_ = 8), and PFDA (*η*_pfc_ = 9) have reported elimination half-lives (t_1/2_) ranging from 3 to 12 years [20,22,23]. Whereas short-chain PFAS, such as perfluorobutanoic acid (PFBA) (*η*_pfc_ = 3) and perfluorobutanesulfonic acid (PFBS) (*η*_pfc_ = 4), have half-lives of 3 and 26 days [21], respectively. PFAS exhibit species-specific t_1/2_, with human having a much higher t_1/2_ than mouse or rat [24]. In addition to renal reabsorption [25], it is postulated that t_1/2_, in part, is dictated by PFAS retention in plasma via binding to plasma proteins, such as albumin. Albumin has been shown to be the major carrier of several PFAS in the body regardless of species, with PFAS binding to albumin in rat and human being similar in strength [26,27]. Although the role of albumin has been studied, few studies have consistently evaluated plasma and albumin binding in a systematic and thorough manner.

Bioaccumulation models that incorporate PFAS protein and lipid binding have been put forward by several groups [28,29,30]. While these simulations provide mechanistic insights into PFAS interactions with biological matrices, they lack accurate physiologically-relevant plasma protein binding data. In assessing xenobiotic binding to plasma, a fraction unbound (f_u_) is usually derived to determine the unbound xenobiotic concentration available to interact with molecular targets, which elicits toxic effects. Plasma protein binding is also a direct parameter to calculate t_1/2_, as compounds that are bound to plasma are unavailable to be distributed to and eliminated by the kidney or liver [31]. It is assumed that the extent of PFAS binding to plasma proteins is influenced by physicochemical properties (e.g., pKa, functional head-group and logD) [32,33]. Several computational models and fluorescence quenching methods have been utilized to determine PFAS binding to albumin, but these methods are indirect and may be limited in their precision and accuracy for several highly bound PFAS [34,35,36,37]. Other direct methods, such as ultrafiltration and equilibrium dialysis, have reported protein binding of PFAS. But many of these studies used bovine serum albumin (BSA) and human serum albumin (HSA), rather than physiologically relevant plasma or serum [27,38,39,40].

Equilibrium dialysis is the current industry-standard methodology to assess protein binding [32]. A challenge for accurately determining PFAS binding is high binding affinity, which can make quantification difficult, and can confound data interpretation [39,40,41]. Therefore, a robust and accurate method, which is broadly applicable for f_u_ derivation, is needed to advance mechanistic understanding of PFAS and evaluate species differences. Herein, a validated presaturation equilibrium dialysis method developed for highly bound compounds [42,43,44] was utilized to derive f_u_ for 14 PFAS with diverse chain lengths (*η*_pfc_ = 3–11) and functional head groups. f_u_ values were measured for both albumin and plasma for four species/strains (CD-1 and C57BL/6 mice, Wistar-Han rats, and humans). All f_u_ comparison combinations were assessed and evaluated against inter-species and inter-matrix correlations and equivalence. Overall, the aim of the work herein was to establish physiologically relevant f_u_ values that can be utilized as more accurate input parameters to model toxicokinetics and understand human translation. Furthermore, the findings described in this manuscript provide essential data to elucidate the mechanisms that drive bioaccumulation and overall elimination of PFAS for rodents and humans with the goal of more physiologically accurate functional translation.

## 2. Materials and Methods

### 2.1. Chemicals and Reagents

The molecular structures of the 14 PFAS evaluated are in Figure S1 and physiochemical properties, such as molecular weight (MW) and logD shown in Table S1. The following fourteen PFAS were either obtained from Accustandard, Inc. (New Haven, CT, USA) or Sigma-Aldrich (St. Louis, MO, USA): Perfluorobutanoic acid (PFBA), perfluoropentanoic acid (PFPA), perfluorohexanoic acid (PFHxA), perfluoroheptanoic acid (PFHpA), perfluorooctanoic acid (PFOA), perfluorononanoic acid (PFNA), perfluorodecanoic acid (PFDA), perfluoroundecanoic acid (PFUDA), perfluorododecanoic acid (PFDoDA), perfluorohexanesulfonic acid (PFHxS), perfluorooctanesulfonamide (PFOSA), 6:2 fluorotelomer sulfonate (6:2 FtS), perfluorobutanesulfonic acid (PFBS), and perfluorooctanesulfonic acid (PFOS). Human, rat (Wistar-Han), and mouse (CD-1; C57BL/6) equal sex pooled (minimum of 3 male and 3 female) plasma was obtained from BioIVT (Hicksville, NY, USA). The equilibrium dialysis device (HTD96) and cellulose membranes with a molecular weight cutoff of 12–14 kDa were obtained from HTDialysis, LLC (Gales Ferry, CT, USA). Human, rat, and mouse albumin, along with all other reagents were obtained from Sigma-Aldrich unless specified otherwise.

### 2.2. Pre-Saturation Equilibrium Dialysis

The methods used herein to determine PFAS binding to plasma and albumin binding were comparable with those previously reported [42,43,44]. The cellulose membranes were hydrated first in deionized (DI) water for 15 min, then transferred to 30% ethanol/DI water for 15 min, and then submerged in Dulbecco’s phosphate-buffered saline (DPBS) for at least 15 min prior to the experiment. The equilibrium dialysis device (EQD) HTD96 (Gales Ferry, CT, USA) was assembled according to manufacturer instructions. DPBS buffer solutions were prepared by adding PFAS with a concentration of either approximately five-fold of the estimated f_u_ values based on initial available data or assumed to be 0.001 if no data was available. The dialysis apparatus and membranes assembled were pre-saturated (250 µL) three times in total with PFAS spiked DPBS buffer solution; twice for 1 h followed by a final 18-h pre-saturation. This same solution was used on the receiver side of the EQD. Species specific matrices with 4% albumin in DPBS solution were prepared for human serum albumin (HSA), rat serum albumin (RSA), and mouse serum albumin (MSA), as well as species specific plasma in human, rat (Wistar-Han), and mouse (CD-1 and C57BL/6). This 4% albumin was used in the albumin binding study to recapitulate plasma concentration, as human albumin concentration in plasma is 3.5–4.5 g/dL (530–680 µM) [45]. The matrix was diluted five-fold using DPBS for PFAS that had preliminary f_u_ values lower than 0.01. Dimethylsulfoxide (DMSO) stock solutions of test PFAS were prepared at 500 μM and added to species-specific plasma or 4% albumin to a final concentration of 5 μM with 1% DMSO. Next, 150 μL of PFAS spiked DPBS was added to the receiver side of the membrane and 150 μL of compound spiked matrix was added to the donor side. The molar ratio between PFAS:albumin avoids any saturation related effects. The HTD apparatus was sealed with a gas permeable membrane (Sigma-Aldrich; Z380059-1PAK) and placed onto an orbital shaker (200 rpm) in a CO2 incubator (5% CO_2_/air, 75% relative humidity) for 18 h at 37 °C. Each PFAS:matrix combination was assessed in quadruplicate. For sample collection, 15 μL of buffer and 45 μL of matrix were sampled from the dialysis device into a 96-deep well plate and matrix-matched with either blank buffer or matrix. The samples were then quenched with 200 μL of cold acetonitrile containing internal standard (tolbutamide 30 nM and carbamazepine 12 nM) to precipitate proteins. Next, the plates were sealed and placed on a vortex mixer for 1 min, and then centrifuged (Beckman Coulter, Fullerton, CA, USA) at 3000 rpm for 5 min at room temperature. Lastly, the supernatant was transferred to a new 96-well plate, fully dried down under nitrogen gas, reconstituted with 200 µL of 50/50 (*v*/*v*) HPLC grade water/acetonitrile, and vortexed for 1 min prior to LC-MS/MS analysis.

### 2.3. LC-MS/MS Quantification

LC-MS/MS analyses were performed on a SCIEX Triple Quad 5500 mass spectrometer (SCIEX, Concord, ON, Canada) equipped with Turbo Ion Spray interface. The HPLC systems consisted of an CTC PAL autosampler (LEAP Technologies, Morrisville, NC, USA) equipped with a model 1290 binary pump (Agilent, Santa Clara, CA, USA). All instruments were controlled and synchronized by SCIEX Analyst software (version 1.6.3). Mobile phases compositions were 0.1% formic acid in water (mobile phase A) and 0.1% formic acid in acetonitrile (mobile phase B). The gradient for PFAS was maintained at 10% B for 0.8 min, followed by a 0.6-min linear increase to 95% B, and kept at 95% B for 4 min before a linear decrease to 10% in 0.1 min. The column was equilibrated at 5% B for 0.5 min before injecting samples. The total run time for each injection was 6 min. The chromatographic separation was conducted on a Phenomenex Kinetex C18 100Å 30 × 2.1 mm column (Torrance, CA, USA) with a flow rate of 0.5 mL/min. The injection volume for each sample was 5 µL. Quadrupoles Q1 and Q3 were set on unit resolution and mass over charge (*m*/*z*) of the analytes and are shown in Table S2. Multiple-reaction-monitoring (MRM) mode using specific precursor/product ion transitions was used for quantification. Data processing was performed using SCIEX Analyst software (version 1.6.3).

### 2.4. Unbound Fraction Calculations

The calculations for f_u_ are shown in Equations (1) and (2), where D is the dilution factor and $f_{u,d}$ is the diluted f_u_ shown below [46]. All calculations are based on area ratios (analyte peak area/IS peak area), and MS responses used for calculations were all in linear ranges. Stability and recovery values for each experiment were run in parallel and passed validation criteria of greater than 70%.

(1) $$Diluted\;f_{u_{d}}=\frac{Receiver\;Area\;Ratio}{Donor\;Area\;Ratio}$$

(2) Undiluted fu=1/D1fu,d−1+1D

### 2.5. Scalar Determination and Statistical Data Analysis

Based on previous publications [46,47], geometric mean f_u_ values were computed for every species, tissue, and compound combination and analyzed on the log scale. A scatterplot matrix was generated to visually assess the relationships between each pairwise combination of species and tissue. These relationships were numerically assessed using Spearman’s rank correlation, the mean absolute fold difference (MAFD), and the geometric mean fold differences. Statistical equivalence within a 2-fold threshold was determined by applying the two one-sided tests (TOST) average bioequivalence procedure to pairwise combination of species and tissue [48,49]. Additionally, the ℓ-correction [50] was applied to adjust the confidence intervals and *p*-values to maintain a family-wise error rate of 0.05 in the presence of 21 total comparisons (all pairwise comparisons of the 7 matrix combinations). This adjustment is designed specifically for multiplicity correction of TOST *p*-values when all pairwise combinations are considered. The same analysis was applied separately to the data in the 3% and 4% albumin cases. The data were analyzed using *R* [51] and the plots were created via the ggplot2 package [52].

## 3. Results

*Species Dependence for PFAS Plasma and Albumin f_u_*

**Figure 1:**
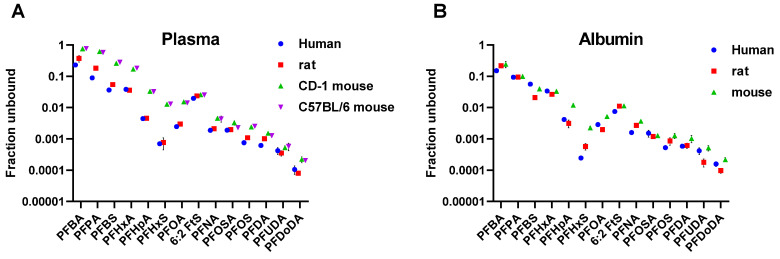
Fraction unbound (f_u_) values for 14 PFAS for human, rat, and mouse plasma (**A**) and albumin (**B**) in binding assays plotted against ascending molecular weight (MW) of the PFAS.

The plasma and albumin binding for 14 PFAS (9 perfluoroalkyl carboxylates, 3 perfluoroalkyl sulfonates, 1 perfluoroalkyl sulfonamide, and 1 fluorotelomer sulfonate) and three species (i.e., human, rat, and mouse) was determined by equilibrium dialysis. The carbon chain lengths ranged from 4 to 12, MW ranged from 214 to 614 Da and logD derived from internal data ranged from 2.23 to 8.54. The f_u_ for the PFAS evaluated was a wide range, from 0.0000796 to 0.77 across the entire data set, demonstrating several orders of magnitude difference in binding (Figure 1). For most PFAS, f_u_ was lower for plasma compared to albumin, indicating that binding affinity was higher in plasma than albumin. The full dataset of individual f_u_ values and coefficient of variation (CV) values are summarized in Table 1.

**Figure 2:**
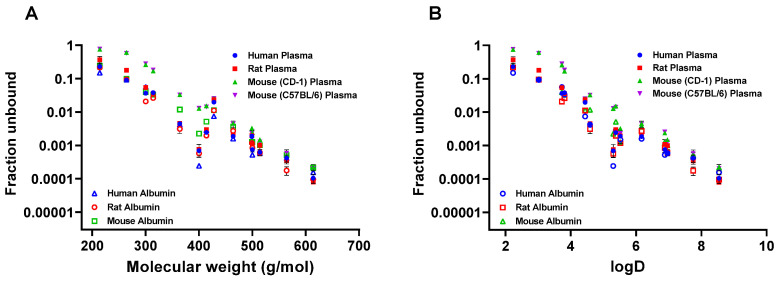
Fraction unbound (f_u_) for 14 PFAS using human, rat, and mouse (CD-1 and C57BL/6) plasma and albumin. (**A**) f_u_ versus molecular weight (MW) (g/mol) and (**B**) f_u_ versus logD.

Next, the relationship between f_u_ and molecular weight or logD were compared (Figure 2A,B). A highly concordant inverse correlation was observed for PFAS plasma or albumin binding and MW or logD (Figure 2). The R2 values for a semi log linear fit (X is linear, Y is log) had a range of 0.928 to 0.99 for all f_u_ values against MW, and a range of 0.913 to 0.996 against logD.

**Figure 3:**
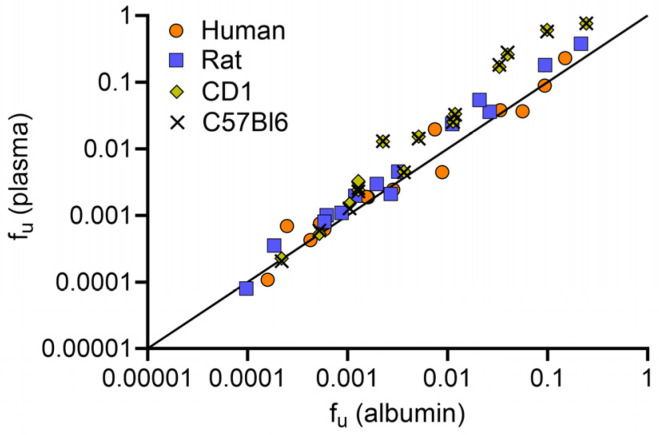
Fraction unbound (f_u_) of plasma and albumin for human, rat, and mouse CD-1 and C57BL/6. The black line represents 1:1 agreement, with f_u_(plasma) = 0.97 × f_u_(albumin) − 0.06, R^2^ = 0.94; SD = 0.25.

Next, the association between plasma and albumin for the 14 PFAS was compared (Figure 3). For human, rat, and mouse, the f_u_ for plasma and albumin were highly concordant. Notably, the f_u_ for PFHxS was lower than expected and did not follow the same trend as the other PFAS.

**Figure 4:**
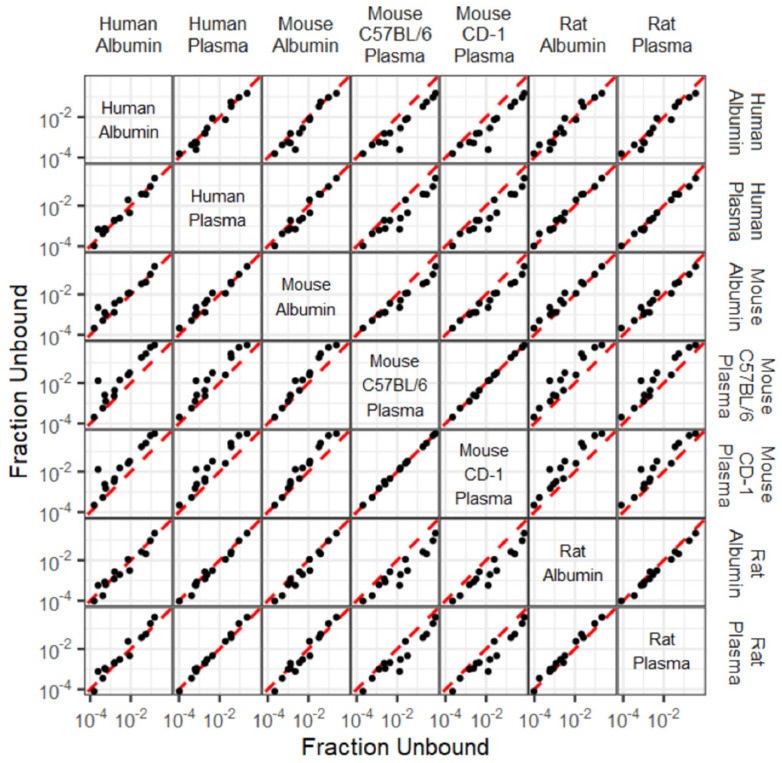
Pairwise comparison of f_u_ for human, rat, and mouse plasma and albumin. Dotted red lines represent 1:1 agreement.

Figure 4 illustrates pairwise comparisons for each pair of albumin or plasma against human, rat (Wistar-Han), and mice (CD-1; C57BL/6) for all 14 PFAS. Comparisons indicate a strong correlation between f_u_ determinations per compound across species and matrix types.

**Figure 5:**
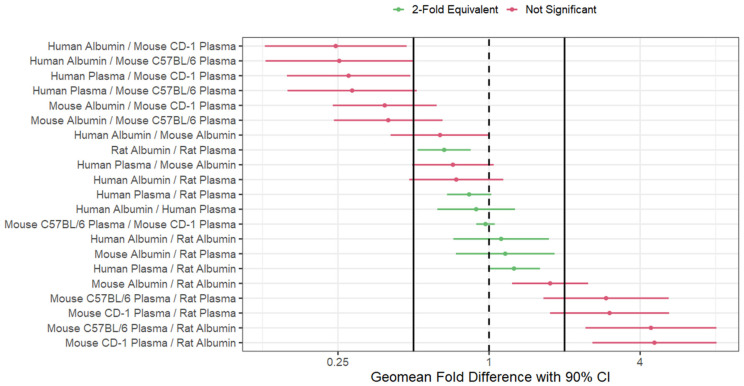
ℓ-correction adjusted TOST equivalence tests conducted for within-matrix pairwise comparisons f_u_ values for all species and plasma/ albumin combinations organized from least to highest geomean fold difference. The dashed line represents a fold difference of 1. The 2-fold difference thresholds were employed to determine equivalence accounting for established assay variance and are shown as black lines (0.5 and 2).

Next, to determine whether plasma and albumin are essentially equivalent matrices, ℓ-correction adjusted two one-sided test (TOST) equivalence tests were run against within-matrix pairwise comparisons for all f_u_ values (Figure 5). The mean absolute fold difference (MAFD) for all combinations ranged from 1.09 to 7.22, and the Spearman rank correlations among all comparisons ranged from 0.92 to 1.00. Individual MAFD, geometric mean fold difference, and Spearman rank correlation values per combination comparison is shown in Table S3. Of the 21 combination equivalence tests conducted, seven matrix combinations showed 2-fold equivalence (rat albumin: rat plasma; human plasma: rat plasma; human albumin: human plasma; mouse C57BL/6 plasma: mouse CD-1 plasma; human albumin: rat albumin; mouse albumin: rat plasma; human plasma: rat albumin). The equivalence test results were also ordered by the specific matrix in the numerator of the fold difference (Figures S2 and S3) and ordered by fold differences on ratios where the pairwise matrix combinations were ordered such that the fold difference was always greater than 1. When comparing species dependent albumin: plasma f_u_ values, human albumin: human plasma and rat albumin: rat plasma was 2-fold equivalent. Within mouse albumin against both C57BL/6 and CD-1 plasma were not equivalent, and MAFD values were above 3. When considering species independence of plasma f_u_ values, human to rat and CD-1 to C57BL/6 showed 2-fold equivalence with an MAFD of 1.32 and 1.09, respectively. However, human to mouse and rat to mouse comparisons both showed MAFD values greater than 4 across different subspecies of mouse suggesting non-equivalence.

Albumin concentrations for rodents have been reported in the literature as rat: 3.0 g/dL; CD-1 mice: 2.7 g/dL; C57BL/6 mice: 2.9 g/dL [53]. Therefore, supplemental analysis of albumin binding for rodents were evaluated at a concentration of 3% albumin to compare physiologically relevant albumin concentrations against other matrices in this study. The values of 3% albumin f_u_ values are shown in Table S4 and did not significantly change the range of f_u_ values within our study. Further pairwise f_u_ comparisons and equivalency tests substituting the 3% albumin concentrations for rodents against the 14 PFAS are shown in Figures S4 and S5 respectively.

## 4. Discussion

With 10,000+ PFAS identified, there is an overwhelming need to predict bioaccumulation patterns in humans using vetted, high-throughput assays for in vitro–in vivo extrapolation. Plasma binding values are critical in measuring free PFAS concentration within the blood compartment of preclinical species and humans. It is a necessary parameter for the assessment of t_1/2_, pharmacokinetic-pharmacodynamic (PKPD) relationships, physiological-based pharmacokinetic modeling (PBPK), and drug–drug interactions (DDI) [31,54]. Within the PFAS space, rodents offer a more comprehensive dataset on toxicokinetics compared to other species [24]. In light of this, our study integrates both rodent and human plasma and albumin to compare protein binding across multiple species.

Several studies have examined protein binding for a handful of PFAS using equilibrium dialysis. A limitation to those studies is that binding was BSA or HSA were used instead rather than whole serum or plasma, which are more relevant matrices than isolated protein [38,39,40]. A study by Ohmori, et al. [55] compared rat plasma binding of different perfluorocarboxylic acids (PFHpA, PFOA, PFNA, and PFDA) by an ultrafiltration method and observed greater than 98% were bound. However, the study did not quantitatively distinguish binding values for each PFAS. This is likely attributed to the exceptionally strong binding properties of these PFAS, which pose a challenge to obtain precise measurements using ultrafiltration due to high nonspecific binding to the device [56]. Historically, methodologies concerning protein and tissue binding have hindered the measurement of f_u_ below 1% (f_u_ < 0.01), largely attributed to FDA regulatory concerns due to low experimental confidence [57]. Yet, recent progress within the field has increased accuracy to measure f_u_ below 1% [42,43,44,58]. Employing an innovative pre-saturation equilibrium dialysis technique used for the development of pharmaceuticals, PFAS f_u_ values for 14 PFAS were obtained in this study, with f_u_ as low as 0.0000796 (bound%: 99.992). With this newly optimized presaturation method, this study surpassed prior limitations to accurately measure protein binding for physiochemically diverse PFAS.

**Figure 6:**
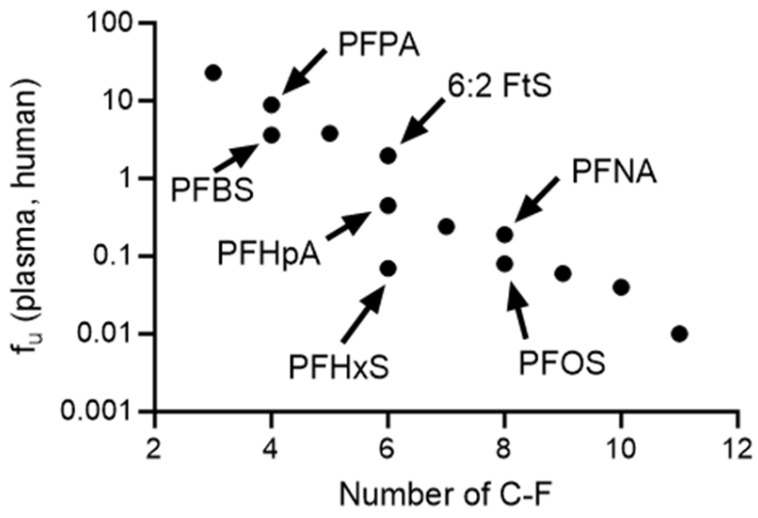
Fractions unbound in human plasma against the number of perfluorinated carbons of the PFAS. PFAS with similar number of perfluorinated carbons but different functional group (carboxylic vs. sulfonic acids) are highlighted in the figure.

Established structural and physiochemical properties of a compound highly influences its ability to bind to plasma proteins [59,60]. Particularly, an increase in lipophilicity is generally associated with increased plasma protein binding [61]. All of the tested PFAS in this set are acids [62], which are expected to bind primarily to albumin compared to other plasma proteins within the blood such as lipoproteins or globulins [63,64]. Several PFAS within this set have high logD values, and as lipophilicity increases, a decrease in f_u_ was observed for all species in both plasma and albumin. logD was also positively correlated with the number of fluorinated carbons of PFAS. This observation could potentially elucidate the higher binding affinity of longer-chained PFAS to plasma proteins. This is consistent with the findings observed by Alesio et al. [40] using BSA. The outcomes revealed a similar trend wherein the longer chained carboxylate, PFDA (C10), was more bound than shorter chained, PFOA (C8), and the longer chained sulfonate, PFOS (C8), was more bound than the shorter chained, PFBS (C4). Increased association constants (K_a_) of PFAS to BSA were observed as carbon chain length increased [38]. Similarly, a decreasing trend was seen for dissociation constants (K_d_) of PFAS against HSA when chain length was increased [65,66]. Within this study, carboxylates with a lower logD were less bound than sulfonates when they have the same number of fluorinated carbon chains. For example, carboxylates, PFPA (*η*_pfc_ = 4; logD: 3.02) and PFNA (*η*_pfc_ = 8; logD:6.17), were less bound than their fluorinated carbon chain respective sulfonates, PFBS (*η*_pfc_ = 4; logD: 3.73) and PFOS (*η*_pfc_ = 8; logD: 6.88) (Figure 6), for all species matrix combinations in this study.

This suggests that logD is an important underlying determinant for protein binding influenced by both fluorinated carbon chain-length and different functional headgroups. Notably, PFHxS (C6; logD: 5.3) had lower f_u_ values for both human and rat plasma compared to PFOS (C8; logD: 6.88). PFHxS is an interesting outlier as it has also shown a longer t_1/2_ than PFOS in human but not in rat [20,24]; suggesting species specific mechanisms may be involved in elimination.

It has been reported that within plasma, the main binding protein against PFOA in human and rat plasma is albumin [27]. It has been further illustrated that albumin is the major carrier protein for PFOS, PFOA, PFHxS, PFNA, and PFDA in native human plasma [26]. Moreover, in whole blood once absorbed, PFAS such as PFOA, PFOS, and PFHxS are not found intracellularly and do not attach to red blood cells [67]. Within this study, plasma and albumin (4%) f_u_ values were equivalent to each other for both human and rat. Physiologically relevant rat albumin (3%) concentrations against rat plasma, although not 2-fold equivalent, yielded an MAFD below 2, suggesting high concordance to each other. Further mechanistic evaluation against species differences of RSA and HSA against PFOA binding by Han et al. [27] showed K_d_ and number of binding sites to be similar between these two species. For our PFAS set, human albumin and rat albumin show equivalent f_u_ values when equal protein concentrations are used. This further validates that there were no species differences when comparing human and rat in both their plasma or albumin f_u_ values within this PFAS set and albumin is the main binding protein within plasma. Similarly, binding values of several drug development compounds within the pharmaceutics space have shown high correlation between human and rat plasma against respective albumin [68].

This equivalence however is not observed when mouse plasma of either strains (CD-1; C57BL/6) is compared with human or rat plasma. Moreover, the albumin f_u_ values of mouse against human or rat albumin also show a lack of equivalence. This may be due to protein structural differences leading to different binding pockets for these PFAS against mouse albumin protein. Unlike tissue homogenate or cell binding which are species independent [33,46,47,69,70], plasma protein binding is reported to be species dependent [31,68,71] which makes using a surrogate species for plasma challenging. It has been suggested that, although not as common, large differences in protein binding can occur between species, observing variances in plasma protein binding of human to mouse reaching fold differences as high as ~17 for some compounds [68]. Although amino acid sequences for albumin across distinct species are similar to human, there are species dependent differences. With 73% of the amino acid sequence of albumin conserved in rat and 72% in mouse, implying specific binding sites may vary leading to differences in protein binding values in plasma [72,73]. Notably, although f_u_ values of mouse matrices lacked equivalence, the Spearman rank correlations for all combinations were high with R2 values above 0.92. This indicates that the individual physiochemical properties per PFAS dictated their binding ability more so than specific binding matrix.

**Figure 7:**
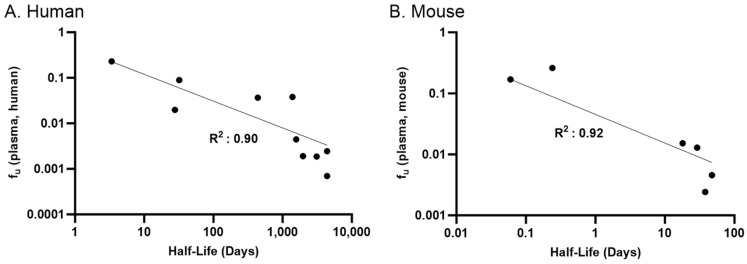
Linear relationship between f_u_ in human and mouse plasma measured in this study and elimination half-lives for PFAS observed in humans (**A**) and mice (**B**).

The plasma f_u_ values of the different mouse strains (CD-1; C57BL/6) were equivalent to each other. However, when comparing plasma for both strains of mice against mouse albumin f_u_ values, they were not equivalent in either the 3% or 4% albumin concentrations with an MAFD greater than ~3 for all comparisons. Interestingly, our PFAS set was more highly bound to mouse albumin than its corresponding plasma which may suggest endogenous compounds (e.g., fatty acids and bilirubin) within the mouse plasma competing for the binding pockets against PFAS [74,75]. Protein binding plays a significant role in overall clearance as it influences the amount of PFAS available. When a compound is bound to plasma proteins, they are unavailable for excretion, potentially leading to slower elimination and longer t_1/2_ [31,61]. Our data supports this, as f_u_ in human plasma is inversely correlated with observed elimination half lives in humans (Figure 7).

Most PFAS generally show high resistance against biotransformation in humans and rodents [76] and are eliminated unchanged by biliary and renal clearance pathways for several different species [77,78,79,80,81]. The f_u_ of PFAS and their respective t_1/2_ in humans are positively correlated (Figure 7). This trend can be seen for both carboxylates and sulfonates. Specifically, for carboxylates, PFBA with a human plasma f_u_ (f_u_, hp) value of 0.23 reports a t_1/2_ of 3.1 days, while PFOA (f_u_, hp: 0.0025) reports a t_1/2_ of 2.3–5.4 years. This trend is also seen for sulfonates as PFBS (f_u_, hp: 0.037) has a t_1/2_ of 26 days, while the more highly bound PFOS (f_u_, hp: 0.00075) has a t_1/2_ of 3.3–5.4 years [24]. These comparisons suggest that the high binding nature of PFAS may be the major driver of their strong accumulation and long t_1/2_ in most species. Previous studies have shown sex-dependence in rat for PFAS half-life, with females showing a significantly shorter half-life than males [22,80,81,82]. However, Han et al. [27] have shown PFOA’s underlying binding to RSA or HSA was sex-independent. Therefore, utilizing pooled plasma and purified albumin is not expected to affect our overall results.

Although protein binding plays a significant role in clearance, with several species and even sex differences for half-life against different PFAS, other mechanisms of elimination of PFAS need to be considered in conjunction with protein binding. It has been reported PFOA and PFOS undergo a high rate of enterohepatic reabsorption [77]. Transporter activity such as Organic anion transporter (OAT) [83,84] and Organic anion transporting polypeptides (OATP) [85], permeability, as well as tissue binding all need to be considered to fully elucidate the toxicokinetics of PFAS.

## 5. Conclusions

The investigation herein evaluated plasma protein binding across mice, rats, and humans for 14 PFAS and compared species and matrix differences, providing valuable insights for further bioaccumulation, elimination half-life, and toxicity research. The pre-saturation methodology used in this study is novel in the PFAS field and allowed the generation of f_u_ values robustly below 1% (f_u_ < 0.01). The results herein illustrate similar binding trends among the three species, and also between plasma and albumin. While minimal differences in protein binding affinity were observed for rat and human, PFAS binding affinity was lower in mouse compared to rat and human. Physiochemical descriptors such as logD, were associated with PFAS binding. With an extensive number of PFAS classified as contaminants that persist and accumulate in the environment, exposure in humans can occur over time through contaminated water and food. This research helps elucidate the degree of binding through trends of different PFAS to better understand the environmental impact and toxicity, so that strategies can be developed to better minimize exposure and accumulation of these toxic chemicals.

## Figures and Tables

**Table 1:** Fraction unbound (f_u_) and coefficient of variance (CV) for human, rat, and mouse (CD-1 and C57BL/6) against plasma and 4% serum albumin in PBS [Human (HSA), rat (RSA), mouse (MSA).].

PFAS	Fraction Unbound (f_u_) and Coefficient of Variance (CV)						
	Human Plasma	Rat Plasma	CD-1 Plasma	C57BL/6 Plasma	HSA	RSA	MSA
PFBA	0.229(4%)	0.37(24%)	0.757(6%)	0.767(2%)	0.15(5%)	0.216(3%)	0.24(22%)
PFPA	0.0888(3%)	0.18(2%)	0.609(4%)	0.577(5%)	0.0933(6%)	0.0942(4%)	0.0987(11%)
PFHxA	0.038(6%)	0.0358(7%)	0.169(5%)	0.182(7%)	0.0336(7%)	0.0264(4%)	0.0329(11%)
PFHpA	0.00445(7%)	0.00454(5%)	0.0331(13%)	0.0323(2%)	0.0336(7%)	0.00313(27%)	0.0118(12%)
PFOA	0.00245(4%)	0.00298(3%)	0.0152(5%)	0.0142(4%)	0.00285(6%)	0.00196(3%)	0.00513(5%)
PFNA	0.00187(3%)	0.0021(6%)	0.00455(6%)	0.00438(22%)	0.00285(6%)	0.00271(2%)	0.00364(6%)
PFDA	0.000618(9%)	0.001(9%)	0.0015(4%)	0.00127(9%)	0.000584(8%)	0.000608(19%)	0.00102(26%)
PFUDA	0.000419(24%)	0.000349(20%)	0.000524(15%)	0.00127(9%)	0.000417(23%)	0.000178(28%)	0.000513(19%)
PFDoDA	0.000105(29%)	0.0000796(10%)	0.000222(21%)	0.000205(9%)	0.000157(16%)	0.0000964(16%)	0.000217(15%)
PFBS	0.0365(4%)	0.0542(3%)	0.261(7%)	0.28(5%)	0.056(5%)	0.0208(8%)	0.0396(11%)
PFHxS	0.000695(3%)	0.00076(39%)	0.0128(11%)	0.013(3%)	0.000245(13%)	0.000573(23%)	0.00225(13%)
PFOS	0.000753(3%)	0.00109(4%)	0.0128(11%)	0.00256(8%)	0.000524(13%)	0.000852(25%)	0.00126(19%)
PFOSA	0.0019(8%)	0.00197(4%)	0.00326(15%)	0.0023(3%)	0.00152(24%)	0.00118(11%)	0.00127(11%)
6:2 FtS	0.0197(2%)	0.0236(4%)	0.0255(2%)	0.0253(2%)	0.00746(0%)	0.0111(4%)	0.0113(9%)

## Data Availability

Dataset available on request from the authors.

## Supplementary Materials

The following supporting information can be downloaded at: https://www.mdpi.com/article/10.3390/toxics12040253/s1, Figure S1: Chemical structures of 14 PFAS selected in study; Figure S2: ℓ-correction adjusted TOST equivalence tests conducted for within-matrix pairwise comparisons fu values for all species and plasma/ albumin combinations grouped by numerator; Figure S3: ℓ-correction adjusted TOST equivalence tests conducted for within-matrix pairwise comparisons f_u_ values for all species and plasma/ albumin combinations sorted by geomean fold difference with matrix ratios ordered such that the geomean fold difference is always greater than 1; Figure S4: Pairwise fraction unbound (f_u_) comparison for all species and subspecies for human, rat, and mouse (CD-1 and C57BL/6) plasma and 3% albumin for rodents; Figure S5: ℓ-correction adjusted TOST equivalence tests conducted for within-matrix pairwise comparisons fraction unbound (f_u_) values for all species and plasma/3% albumin combinations organized from least to highest geomean fold difference; Table S1: 14 total PFAS within the study including perfluoroalkyl carboxylates, perfluoroalkyl sulfonates, perfluoroalkyl sulfonamide, and fluorotelomer sulfonate with respective PFAS acronym, full PFAS name, number of carbons within carbon chain, molecular weight (g/mol), and logD; Table S2: PFAS LC-MS/MS multiple reaction monitoring (MRM) conditions including, quadrupoles (Q1, Q3), polarity, declustering potential (DP), and collision energy (CE); Table S3: Species and matrix combination comparisons including mean absolute fold-difference (MAFD), spearman rank-correlation, and geometric mean fold difference; Table S4: Fraction unbound (f_u_) and %CV for species specific 3% serum albumin [rat (RSA), and mouse (MSA)].
