# Supplementary material for: Species-Specific Unbound Fraction Differences in Highly Bound PFAS: A Comparative Study across Human, Rat, and Mouse Plasma and Albumin
Source: Toxics. 2024 Mar 29;12(4):253. doi: 10.3390/toxics12040253 (PMC11054487; doi:10.3390/toxics12040253)
Supplement: Supplementary file 1 [file toxics-12-00253-s001.zip › toxics-2886181-supplementary.pdf]

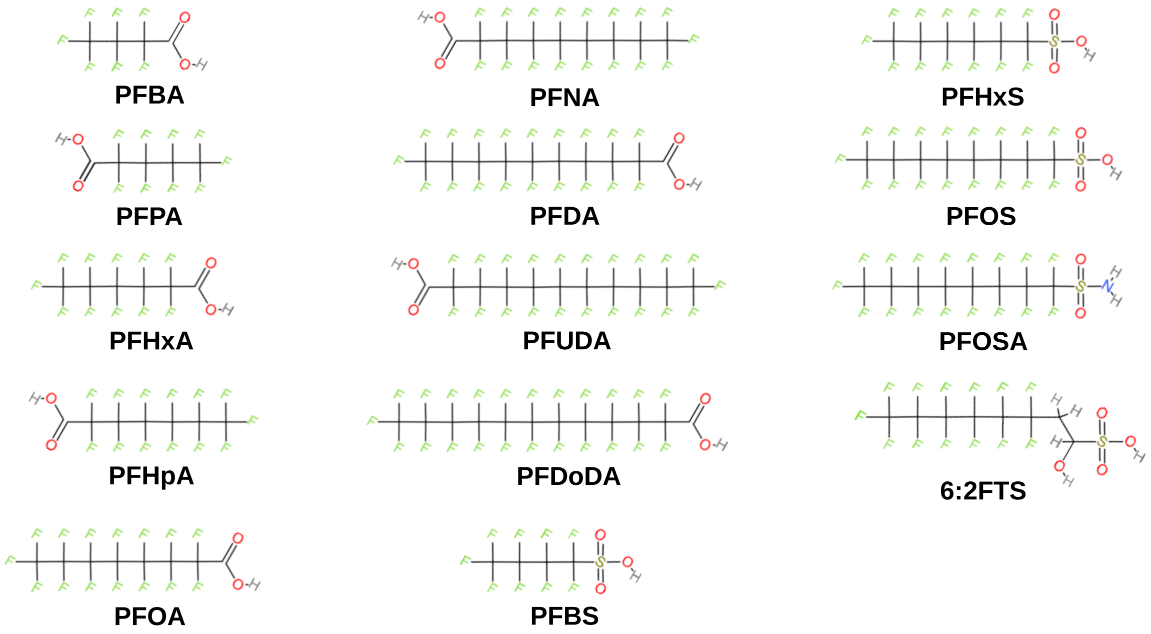


**Supplementary Figure S1.** Chemical structures of 14 PFAS selected in study.

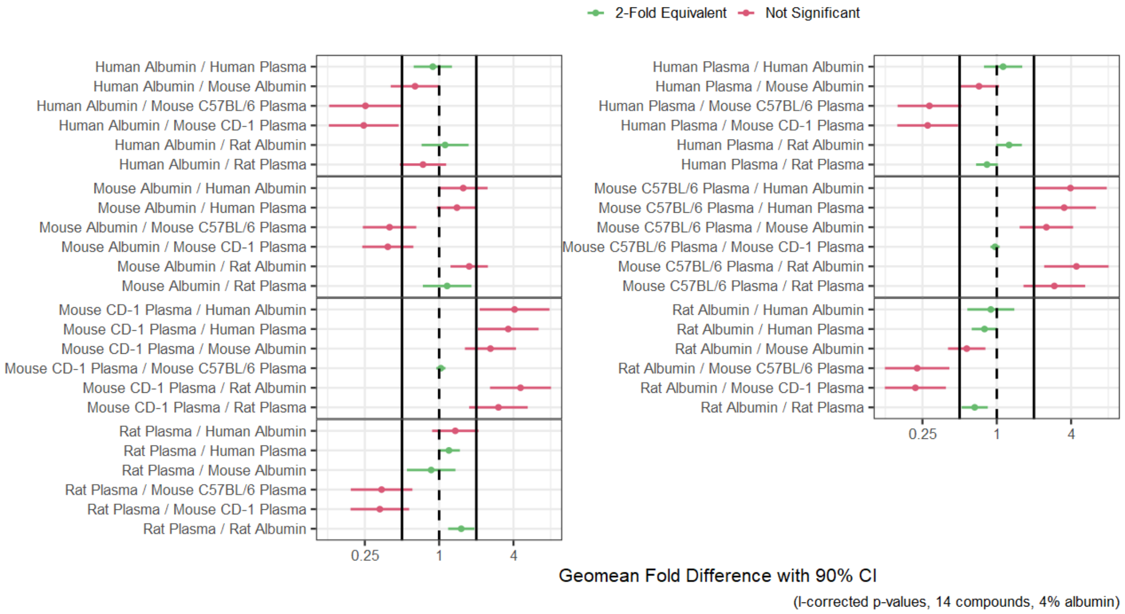


**Supplementary Figure S2.** ℓ-correction adjusted TOST equivalence tests conducted for within-matrix pairwise comparisons fu values for all species and plasma/ albumin combinations grouped by numerator. 2-fold difference threshold employed to determine equivalence accounting for established assay variance.

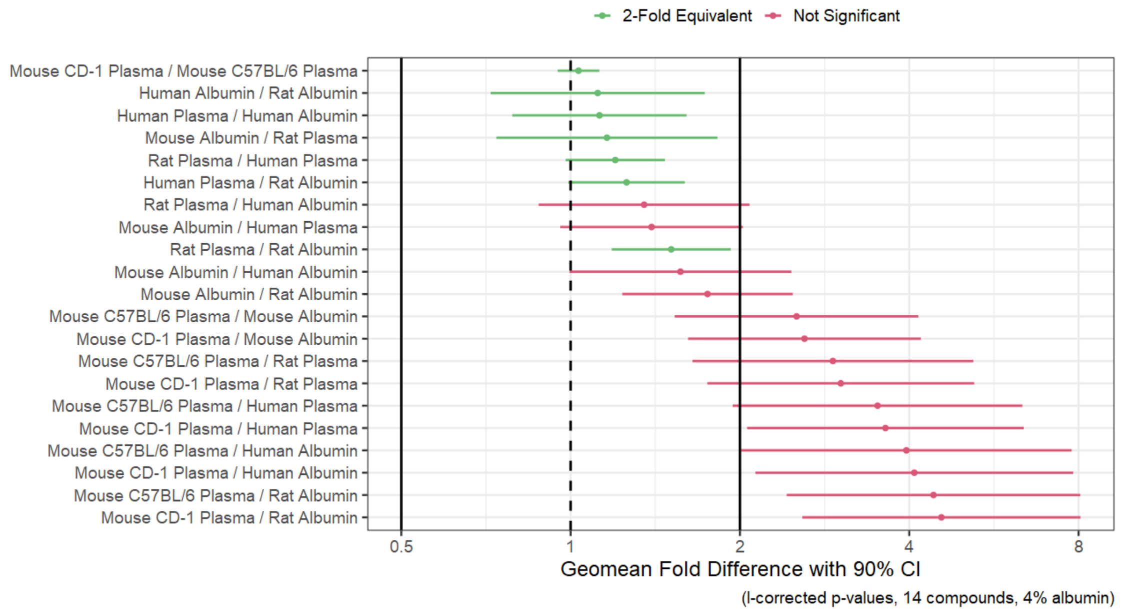


**Supplementary Figure S3.** ℓ-correction adjusted TOST equivalence tests conducted for within-matrix pairwise comparisons fu values for all species and plasma/ albumin combinations sorted by geomean fold difference with matrix ratios ordered such that the geomean fold difference is always greater than 1. 2-fold difference threshold employed to determine equivalence accounting for established assay variance.

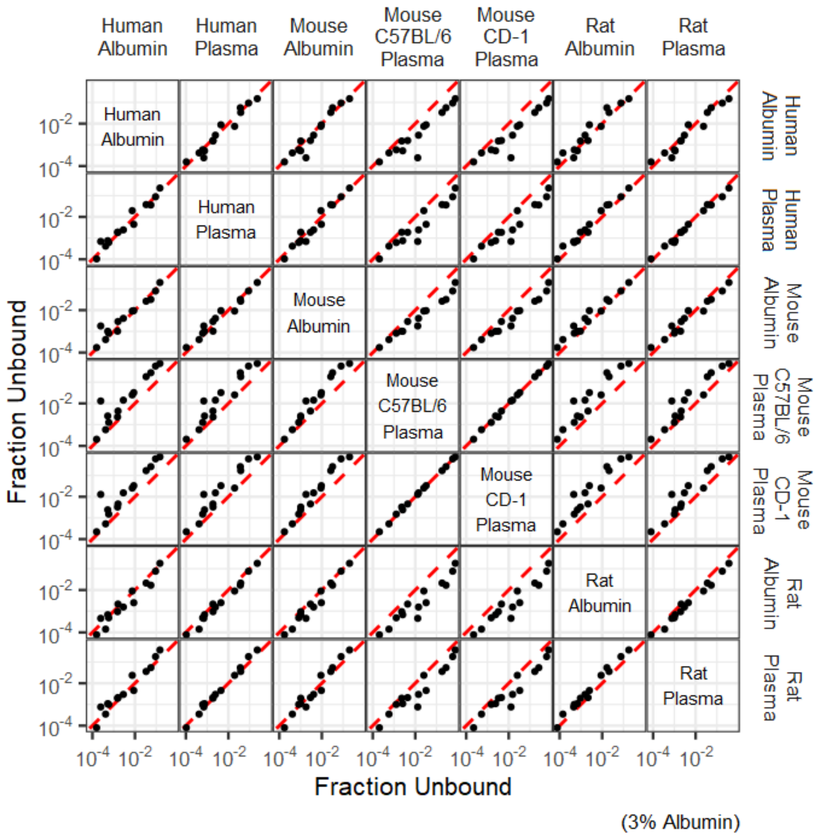


**Supplementary Figure S4.** Pairwise fraction unbound ($f_u$) comparison for all species and subspecies for human, rat, and mouse (CD-1 and C57BL/6) plasma and 3% albumin for rodents.

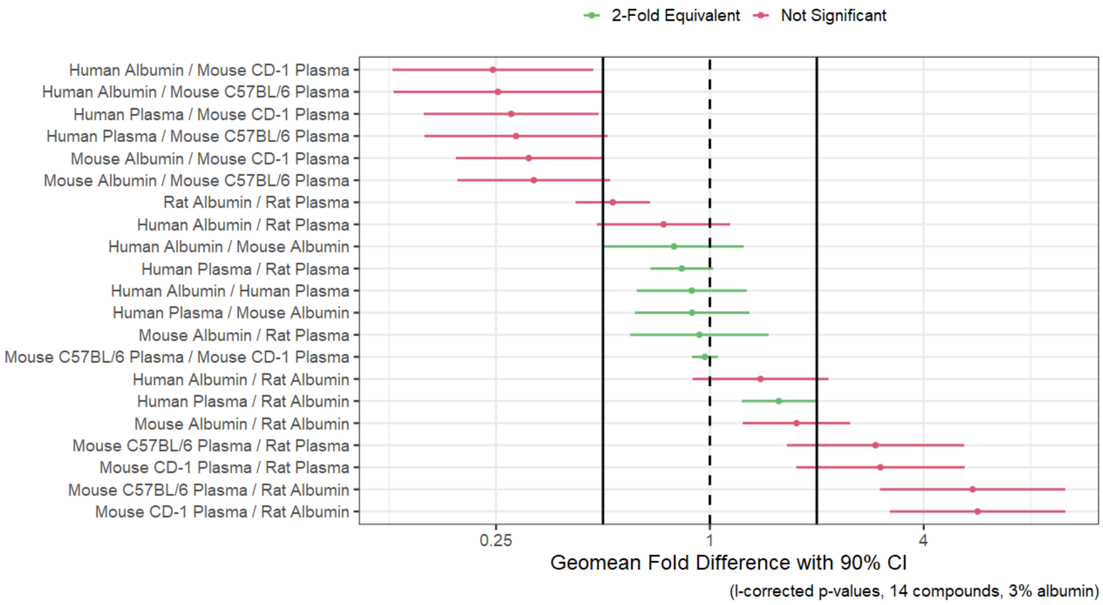


**Supplementary Figure S5.** ℓ-correction adjusted TOST equivalence tests conducted for within-matrix pairwise comparisons fraction unbound ($f_u$) values for all species and plasma/ 3% albumin combinations organized from least to highest geomean fold difference. 2-fold difference threshold employed to determine equivalence accounting for established assay variance.

**Supplementary Table S1.** 14 total PFAS within the study including perfluoroalkyl carboxylates, perfluoroalkyl sulfonates, perfluoroalkyl sulfonamide, and fluorotelomer sulfonate with respective PFAS acronym, full PFAS name, number of carbons within carbon chain, molecular weight (g/mol), and logD.

| PFAS | Full Name | Carbon Chain | Molecular weight | logD |
|---|---|---|---|---|
| Perfluoroalkyl Carboxylates | | | (g/mol) | |
| PFBA | Perfluorobutanoic acid | **4** | 214.04 | 2.23 |
| PFPA | Perfluoropentanoic acid | **5** | 264.05 | 3.02 |
| PFHxA | Perfluorohexanoic acid | **6** | 314.05 | 3.81 |
| PFHpA | Perfluoroheptanoic acid | **7** | 364.06 | 4.59 |
| PFOA | Perfluorooctanoic acid | **8** | 414.07 | 5.38 |
| PFNA | Perfluorononanoic acid | **9** | 464.08 | 6.17 |
| PFDA | Perfluorodecanoic acid | **10** | 514.08 | 6.96 |
| PFUDA | Perfluoroundecanoic acid | **11** | 564.09 | 7.75 |
| PFDoDA | Perfluorododecanoic acid | **12** | 614.1 | 8.54 |
| Perfluoroalkyl Sulfonates | | | | |
| PFBS | Perfluorobutane sulfonic acid | **4** | 300.1 | 3.73 |
| PFHxS | Perfluorohexane sulfonic acid | **6** | 400.12 | 5.30 |
| PFOS | Perfluorooctane sulfonic acid | **8** | 500.13 | 6.88 |
| Perfluoroalkyl Sulfonamides | | | | |
| PFOSA | Perfluorooctanesulfonamide | **8** | 499.14 | 5.52 |
| Fluorotelomer Sulfonates | | | | |
| 6:2 FtS | 6:2 Fluorotelomer sulfonate | **8** | 427.98 | 4.44 |

**Supplementary Table S2.** PFAS LC-MS/MS multiple reaction monitoring (MRM) conditions including, quadrupoles (Q1, Q3), polarity, declustering potential (DP), and collision energy (CE).

| PFAS | MRM transitions | | Polarity | DP | CE |
|---|---|---|---|---|---|
| | Q1 | Q3 | | | |
| PFBA | 212.99 | 169.01 | Negative | -30 | -15 |
| PFPA | 262.99 | 219.02 | Negative | -30 | -15 |
| PFHxA | 312.99 | 269.04 | Negative | -30 | -15 |
| PFHpA | 363.01 | 319.06 | Negative | -30 | -15 |
| PFOA | 413.1 | 369.13 | Negative | -30 | -15 |
| PFNA | 463.04 | 419.15 | Negative | -30 | -15 |
| PFDA | 513.05 | 469.14 | Negative | -30 | -15 |
| PFUDA | 563.08 | 519.16 | Negative | -40 | -15 |
| PFTeDA | 713.03 | 669.09 | Negative | -40 | -15 |
| PFBS | 298.96 | 80.1 | Negative | -80 | -65 |
| PFHxS | 399.07 | 80.1 | Negative | -20 | -55 |
| PFOS | 499.1 | 80.1 | Negative | -120 | -35 |
| 6:2FTS | 427.16 | 81 | Negative | -80 | -65 |
| PFOSA | 498.09 | 169.01 | Negative | -120 | -35 |

**Supplementary Table S3.** Species and matrix combination comparisons including mean absolute fold-difference (MAFD), spearman rank-correlation, and geometric mean fold difference.

| 1st Tissue | 2nd Tissue | Mean Absolute Fold Difference | Spearman's Rank Correlation | Geometric Mean Fold Difference (1st Tissue/2nd Tissue) |
|---|---|---|---|---|
| Human Albumin (4%) | Human Plasma | 1.52 | 0.96 | 0.89 |
| Human Albumin (4%) | Mouse Albumin (3%) | 1.81 | 0.95 | 0.79 |
| Human Albumin (4%) | Mouse C57BL/6 Plasma | 7.22 | 0.92 | 0.25 |
| Human Albumin (4%) | Mouse CD-1 Plasma | 7.21 | 0.93 | 0.24 |
| Human Albumin (4%) | Rat Albumin (3%) | 1.87 | 0.98 | 1.39 |
| Human Albumin (4%) | Rat Plasma | 1.81 | 0.99 | 0.74 |
| Human Plasma | Human Albumin (4%) | 1.52 | 0.96 | 1.13 |
| Human Plasma | Mouse Albumin (3%) | 1.58 | 0.97 | 0.89 |
| Human Plasma | Mouse C57BL/6 Plasma | 4.84 | 0.95 | 0.28 |
| Human Plasma | Mouse CD-1 Plasma | 4.88 | 0.96 | 0.28 |
| Human Plasma | Rat Albumin (3%) | 1.66 | 0.98 | 1.56 |
| Human Plasma | Rat Plasma | 1.32 | 0.99 | 0.83 |
| Mouse Albumin (3%) | Human Albumin (4%) | 1.81 | 0.95 | 1.26 |
| Mouse Albumin (3%) | Human Plasma | 1.58 | 0.97 | 1.12 |
| Mouse Albumin (3%) | Mouse C57BL/6 Plasma | 3.86 | 0.99 | 0.32 |
| Mouse Albumin (3%) | Mouse CD-1 Plasma | 3.90 | 1.00 | 0.31 |
| Mouse Albumin (3%) | Rat Albumin (3%) | 1.96 | 0.96 | 1.75 |
| Mouse Albumin (3%) | Rat Plasma | 1.75 | 0.97 | 0.93 |
| Mouse C57BL/6 Plasma | Human Albumin (4%) | 7.22 | 0.92 | 3.95 |
| Mouse C57BL/6 Plasma | Human Plasma | 4.84 | 0.95 | 3.51 |
| Mouse C57BL/6 Plasma | Mouse Albumin (3%) | 3.86 | 0.99 | 3.13 |
| Mouse C57BL/6 Plasma | Mouse CD-1 Plasma | 1.09 | 1.00 | 0.97 |

| | | | | |
|---|---|---|---|---|
| Mouse C57BL/6 Plasma | Rat Albumin (3%) | 7.71 | 0.93 | 5.49 |
| Mouse C57BL/6 Plasma | Rat Plasma | 4.05 | 0.95 | 2.93 |
| Mouse CD-1 Plasma | Human Albumin (4%) | 7.21 | 0.93 | 4.08 |
| Mouse CD-1 Plasma | Human Plasma | 4.88 | 0.96 | 3.63 |
| Mouse CD-1 Plasma | Mouse Albumin (3%) | 3.90 | 1.00 | 3.23 |
| Mouse CD-1 Plasma | Mouse C57BL/6 Plasma | 1.09 | 1.00 | 1.03 |
| Mouse CD-1 Plasma | Rat Albumin (3%) | 7.73 | 0.94 | 5.67 |
| Mouse CD-1 Plasma | Rat Plasma | 4.08 | 0.95 | 3.02 |
| Rat Albumin (3%) | Human Albumin (4%) | 1.87 | 0.98 | 0.72 |
| Rat Albumin (3%) | Human Plasma | 1.66 | 0.98 | 0.64 |
| Rat Albumin (3%) | Mouse Albumin (3%) | 1.96 | 0.96 | 0.57 |
| Rat Albumin (3%) | Mouse C57BL/6 Plasma | 7.71 | 0.93 | 0.18 |
| Rat Albumin (3%) | Mouse CD-1 Plasma | 7.73 | 0.94 | 0.18 |
| Rat Albumin (3%) | Rat Plasma | 1.97 | 0.99 | 0.53 |
| Rat Plasma | Human Albumin (4%) | 1.81 | 0.99 | 1.35 |
| Rat Plasma | Human Plasma | 1.32 | 0.99 | 1.20 |
| Rat Plasma | Mouse Albumin (3%) | 1.75 | 0.97 | 1.07 |
| Rat Plasma | Mouse C57BL/6 Plasma | 4.05 | 0.95 | 0.34 |
| Rat Plasma | Mouse CD-1 Plasma | 4.08 | 0.95 | 0.33 |
| Rat Plasma | Rat Albumin (3%) | 1.97 | 0.99 | 1.88 |
| Human Albumin (4%) | Human Plasma | 1.52 | 0.96 | 0.89 |
| Human Albumin (4%) | Mouse Albumin (4%) | 2.08 | 0.95 | 0.64 |
| Human Albumin (4%) | Mouse C57BL/6 Plasma | 7.22 | 0.92 | 0.25 |
| Human Albumin (4%) | Mouse CD-1 Plasma | 7.21 | 0.93 | 0.24 |
| Human Albumin (4%) | Rat Albumin (4%) | 1.72 | 0.98 | 1.12 |
| Human Albumin (4%) | Rat Plasma | 1.81 | 0.99 | 0.74 |
| Human Plasma | Human Albumin (4%) | 1.52 | 0.96 | 1.13 |
| Human Plasma | Mouse Albumin (4%) | 1.73 | 0.97 | 0.72 |

| | | | | |
|---|---|---|---|---|
| Human Plasma | Mouse C57BL/6 Plasma | 4.84 | 0.95 | 0.28 |
| Human Plasma | Mouse CD-1 Plasma | 4.88 | 0.96 | 0.28 |
| Human Plasma | Rat Albumin (4%) | 1.40 | 0.98 | 1.26 |
| Human Plasma | Rat Plasma | 1.32 | 0.99 | 0.83 |
| Mouse Albumin (4%) | Human Albumin (4%) | 2.08 | 0.95 | 1.57 |
| Mouse Albumin (4%) | Human Plasma | 1.73 | 0.97 | 1.39 |
| Mouse Albumin (4%) | Mouse C57BL/6 Plasma | 3.12 | 0.99 | 0.40 |
| Mouse Albumin (4%) | Mouse CD-1 Plasma | 3.14 | 1.00 | 0.38 |
| Mouse Albumin (4%) | Rat Albumin (4%) | 1.95 | 0.96 | 1.75 |
| Mouse Albumin (4%) | Rat Plasma | 1.78 | 0.97 | 1.16 |
| Mouse C57BL/6 Plasma | Human Albumin (4%) | 7.22 | 0.92 | 3.95 |
| Mouse C57BL/6 Plasma | Human Plasma | 4.84 | 0.95 | 3.51 |
| Mouse C57BL/6 Plasma | Mouse Albumin (4%) | 3.12 | 0.99 | 2.52 |
| Mouse C57BL/6 Plasma | Mouse CD-1 Plasma | 1.09 | 1.00 | 0.97 |
| Mouse C57BL/6 Plasma | Rat Albumin (4%) | 6.19 | 0.93 | 4.42 |
| Mouse C57BL/6 Plasma | Rat Plasma | 4.05 | 0.95 | 2.93 |
| Mouse CD-1 Plasma | Human Albumin (4%) | 7.21 | 0.93 | 4.08 |
| Mouse CD-1 Plasma | Human Plasma | 4.88 | 0.96 | 3.63 |
| Mouse CD-1 Plasma | Mouse Albumin (4%) | 3.14 | 1.00 | 2.60 |
| Mouse CD-1 Plasma | Mouse C57BL/6 Plasma | 1.09 | 1.00 | 1.03 |
| Mouse CD-1 Plasma | Rat Albumin (4%) | 6.21 | 0.94 | 4.56 |
| Mouse CD-1 Plasma | Rat Plasma | 4.08 | 0.95 | 3.02 |
| Rat Albumin (4%) | Human Albumin (4%) | 1.72 | 0.98 | 0.90 |
| Rat Albumin (4%) | Human Plasma | 1.40 | 0.98 | 0.80 |
| Rat Albumin (4%) | Mouse Albumin (4%) | 1.95 | 0.96 | 0.57 |
| Rat Albumin (4%) | Mouse C57BL/6 Plasma | 6.19 | 0.93 | 0.23 |
| Rat Albumin (4%) | Mouse CD-1 Plasma | 6.21 | 0.94 | 0.22 |
| Rat Albumin (4%) | Rat Plasma | 1.65 | 0.99 | 0.66 |

| | | | | |
|---|---|---|---|---|
| Rat Plasma | Human Albumin (4%) | 1.81 | 0.99 | 1.35 |
| Rat Plasma | Human Plasma | 1.32 | 0.99 | 1.20 |
| Rat Plasma | Mouse Albumin (4%) | 1.78 | 0.97 | 0.86 |
| Rat Plasma | Mouse C57BL/6 Plasma | 4.05 | 0.95 | 0.34 |
| Rat Plasma | Mouse CD-1 Plasma | 4.08 | 0.95 | 0.33 |
| Rat Plasma | Rat Albumin (4%) | 1.65 | 0.99 | 1.51 |

**Supplementary Table S4.** Fraction unbound ($f_u$) and %CV for species specific 3% serum albumin [rat (RSA), and mouse (MSA)].

| PFAS | RSA 3% | MSA 3% |
|---|---|---|
| PFBA | 0.18 (3%) | 0.206 (23%) |
| PFPA | 0.0769 (4%) | 0.0809 (11%) |
| PFHxA | 0.0212 (4%) | 0.0266 (11%) |
| PFHpA | 0.00257 (27%) | 0.00954 (12%) |
| PFOA | 0.00157 (3%) | 0.00411 (5%) |
| PFNA | 0.00217 (2%) | 0.00292 (6%) |
| PFDA | 0.000494 (19%) | 0.000839 (26%) |
| PFUDA | 0.000147 (28%) | 0.000417 (19%) |
| PFDoDA | 0.0000777 (16%) | 0.0001749 (15%) |
| PFBS | 0.0168 (8%) | 0.0321 (11%) |
| PFHxS | 0.00047 (23%) | 0.00181 (13%) |
| PFOS | 0.000697 (25%) | 0.00102 (19%) |
| PFOSA | 0.000952 (11%) | 0.00102 (11%) |
| 6:2 FtS | 0.00892 (4%) | 0.0091 (9%) |
